# Genome-wide analysis captures the determinants of the antibiotic cross-resistance interaction network

**DOI:** 10.1038/ncomms5352

**Published:** 2014-07-08

**Authors:** Viktória Lázár, István Nagy, Réka Spohn, Bálint Csörgő, Ádám Györkei, Ákos Nyerges, Balázs Horváth, Andrea Vörös, Róbert Busa-Fekete, Mónika Hrtyan, Balázs Bogos, Orsolya Méhi, Gergely Fekete, Balázs Szappanos, Balázs Kégl, Balázs Papp, Csaba Pál

**Affiliations:** 1 Synthetic and Systems Biology Unit, Institute of Biochemistry, Biological Research Centre, Temesvari krt 62, Szeged 6726, Hungary; 2 Sequencing Platform, Institute of Biochemistry, Biological Research Centre, Temesvari krt 62, Szeged 6726, Hungary; 3 MTA-SZTE Research Group on Artificial Intelligence, Tisza Lajos krt 103., H-6720 Szeged, Hungary; 4 Linear Accelerator Laboratory, University of Paris-Sud, CNRS, Orsay 91898, France; 5 These authors contributed equally to this work

## Abstract

Understanding how evolution of antimicrobial resistance increases resistance to other drugs is a challenge of profound importance. By combining experimental evolution and genome sequencing of 63 laboratory-evolved lines, we charted a map of cross-resistance interactions between antibiotics in *Escherichia coli*, and explored the driving evolutionary principles. Here, we show that (1) convergent molecular evolution is prevalent across antibiotic treatments, (2) resistance conferring mutations simultaneously enhance sensitivity to many other drugs and (3) 27% of the accumulated mutations generate proteins with compromised activities, suggesting that antibiotic adaptation can partly be achieved without gain of novel function. By using knowledge on antibiotic properties, we examined the determinants of cross-resistance and identified chemogenomic profile similarity between antibiotics as the strongest predictor. In contrast, cross-resistance between two antibiotics is independent of whether they show synergistic effects in combination. These results have important implications on the development of novel antimicrobial strategies.

## Abstract (web summary)

Understanding how evolution of antimicrobial resistance increases resistance to other drugs is of key importance. Here, Lazar *et al.* build a map of cross-resistance interactions between antibiotics in *Escherichia coli* and show that chemical and genomic similarities are good predictors of bacterial cross-resistance.

## Graphical abstract

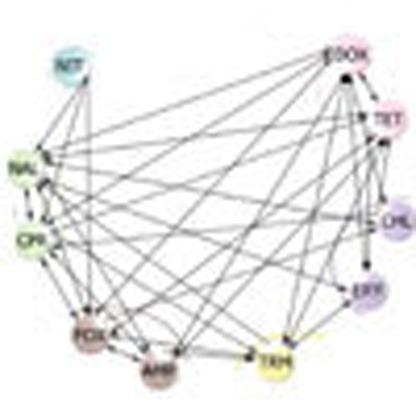

Evolutionary adaptation to a specific environment may result in correlated fitness changes in other environments^1^,^2^. While such evolutionary interactions are widespread in nature, the general principles and underlying molecular mechanisms remain poorly understood^3^. Antibiotic resistance in bacteria offers a platform to systematically investigate evolutionary adaptations. The evolution of antibiotic resistance is frequently mediated by the accumulation of mutations across the genome during therapy^4^. The accumulation of such mutations can potentially change the sensitivity to many antibiotics simultaneously^5^. Despite their clinical relevance, the altered sensitivity profiles of antibiotic resistant strains have not been investigated systematically, except for a pioneering but largely phenomenological study published 60 years ago^6^. Recent works^7^,^8^ investigated the frequency and mechanisms underlying collateral sensitivity (that is, when genetic adaptation under antibiotic stress yields enhanced sensitivity to other antibiotics). The aim of the current paper is to provide insights into the general principles driving cross-resistance interactions. Here, we (i) chart the network of such evolutionary cross-resistance interactions, (ii) explore the underlying molecular mechanisms and (iii) investigate the extent to which cross-resistance is predictable based on the knowledge of antibiotic properties and the set of accumulated mutations.

To accomplish these goals, we initiated parallel laboratory evolutionary experiments with *Escherichia coli* to adapt to increasing dosages of one of 12 antibiotics, and inferred a network of cross-resistance interactions. Laboratory-evolved lines were subjected to whole-genome sequence analysis and biochemical assays to decipher the underlying molecular mechanisms of these interactions.

The following main conclusions were reached. First, the cross-resistance network was dense, indicating that exposure to a single antibiotic frequently yields multidrug resistance. Cross-resistance between two antibiotics is largely independent of whether they show synergistic effects in combination. Second, evolution of resistance is partly achieved through the accumulation of genomic rearrangements and loss-of-function mutations. Third, as parallel evolution at the molecular level is prevalent, cross-resistance patterns are predicable based on the set of accumulated mutations and chemogenomic profile similarities between antibiotics. Taken together, resistance evolution is governed by mutations with highly pleiotropic, but predictable side-effects.

## Results

### High-throughput laboratory evolutionary experiments

In a previous work^7^, we initiated high-throughput laboratory evolutionary experiments starting with *E. coli* K12. Parallel evolving bacterial populations were exposed to 1 of 12 antibiotics (Table 1). Starting from a single ancestral clone, populations were allowed to evolve to successively higher antibiotic concentrations. Evolved populations reached up to 328-fold increases in the minimum inhibitory concentrations relative to the ancestor (Supplementary Table 1). For each antibiotic, 10 independently evolved, resistant populations were subjected to further analysis.

**Figure 1:**
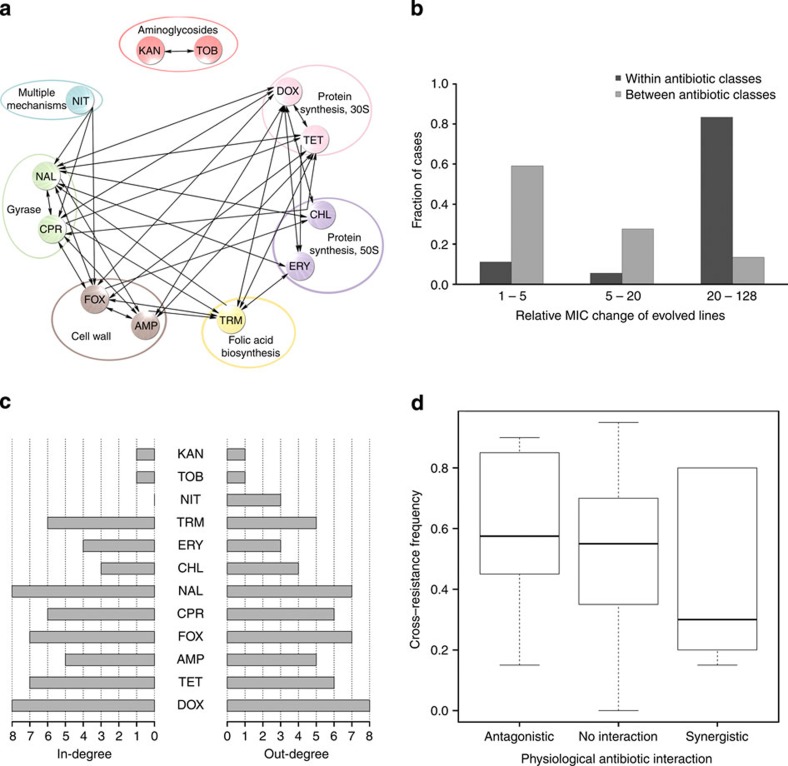
Cross-resistance interactions and their general properties. (**a**) Network of cross-resistance interactions. Antibiotics are grouped according to their mode of action. An arrow from antibiotic A to antibiotic B indicates that adaptation to A decreased sensitivity to B in at least 50% of the evolved populations. (**b**) Distribution of the strength of cross-resistance interactions, as estimated by *E*-tests. (**c**) Cross-resistance interaction degrees of antibiotics. In-degree measures the number of antibiotic treatments which select for increased resistance against a given antibiotic while out-degree is defined as the number of antibiotics to which cross-resistance evolves when adapting to a given drug. The data are based on that of **a**. (**d**) The frequency of cross-resistance interactions between antibiotics is independent of whether they show physiological interactions (that is, synergy or antagonism), *P*=0.35, *N*=45, Kruskal–Wallis test. Aminoglycosides are excluded from the analysis as they show an especially large number of synergistic interactions and are strongly depleted in cross-resistance interactions with other antibiotics). Box plot presents the median and first and third quartiles, with whiskers showing either the maximum (minimum) value or 1.5 times the interquartile range of the data, whichever is smaller (larger).

Using an established high-throughput and highly sensitive protocol^7^, we previously measured the corresponding changes in susceptibilities of the 120 laboratory-evolved populations to all other 11 antibiotics (Supplementary Data 1). The reliability of the detected cross-resistance interactions was confirmed by measuring changes in minimum inhibitory concentrations using standard *E*-tests (Fig. 1b): the rates of false positives and negatives were around 5 and 16%, respectively (Supplementary Data 2). This allowed us to calculate the frequency of cross-resistance (FCR) interactions for each antibiotic pair (see Methods) and ultimately chart a map of cross-resistance between antibiotics (Fig. 1a).

### Properties of the cross-resistance network

Three main patterns emerged from our map (Fig. 1a). First, the evolution of multidrug resistance was frequent under a single antibiotic pressure: on average, 52% of all investigated antibiotic pairs showed cross-resistance in at least one direction. However, the strength of cross-resistance interactions in the data set was highly variable and caused 2 to 128-fold increases in minimum inhibitory concentrations (Fig. 1b). Antibiotic pairs belonging to different functional classes also showed evidence of cross-resistance (Supplementary Data 2). For example, lines adapted to the gyrase inhibitor ciprofloxacin displayed 48 to 68-fold enhancements in resistance to a cell wall inhibitor (cefoxitin).

Second, antibiotics differed in their numbers of cross-resistance interactions (Fig. 1c). For instance, adaptation to doxycycline or fluoroquinolones generally led to multidrug resistance. As expected, the corresponding evolved lines frequently accumulated mutations in putative multidrug resistance genes (see below). In sharp contrast, lines adapted to aminoglycosides had few if any cross-resistance interactions, reflecting unusual resistance mechanisms and a unique pathway for cellular uptake^9^. Next, we investigated the other side of the coin: the extent to which resistance to a given antibiotic was achieved by selection to other antibiotics. For each antibiotic, we calculated the number of different antibiotic treatments that select for increased resistance against a given antibiotic (see in-degree on Fig. 1c). In this case, nitrofurantoin was an interesting outlier: nitrofurantoin resistance was reached in only 3% of the populations adapted to other antibiotics (Supplementary Data 1).

Third, prior works indicated that concurrent application of two antibiotics could be used to counter resistance evolution^10^. The efficiency of such combination treatment is determined by at least two factors. It depends on whether the two antibiotics show a synergistic or antagonistic effect on bacterial growth when used in combination (that is, their combined effect is above or below the sum of their individual effects)^11^. Furthermore, it depends on the availability of mutations that confer resistance to both antibiotics. Therefore, it is important to establish whether the antibiotic cross-resistance map overlaps with results of a previous antibiotic combination screen^12^. Aminoglycosides displayed an especially large number of synergistic interactions on growth when used in combination with other antibiotics and, as noted above, were also depleted of cross-resistance with other antibiotic classes (*P*=0.008, *N*=55, Kruskal–Wallis test). After excluding this antibiotic class, neither synergistic nor antagonistic antibiotic pairs were enriched in cross-resistance interactions (*P*=0.35, *N*=45, Kruskal–Wallis test; Fig. 1d). Thus, networks based on evolutionary and physiological antibiotic interactions show little overlap.

### Adaptive mutations dominate in the laboratory-evolved lines

**Figure 2:**
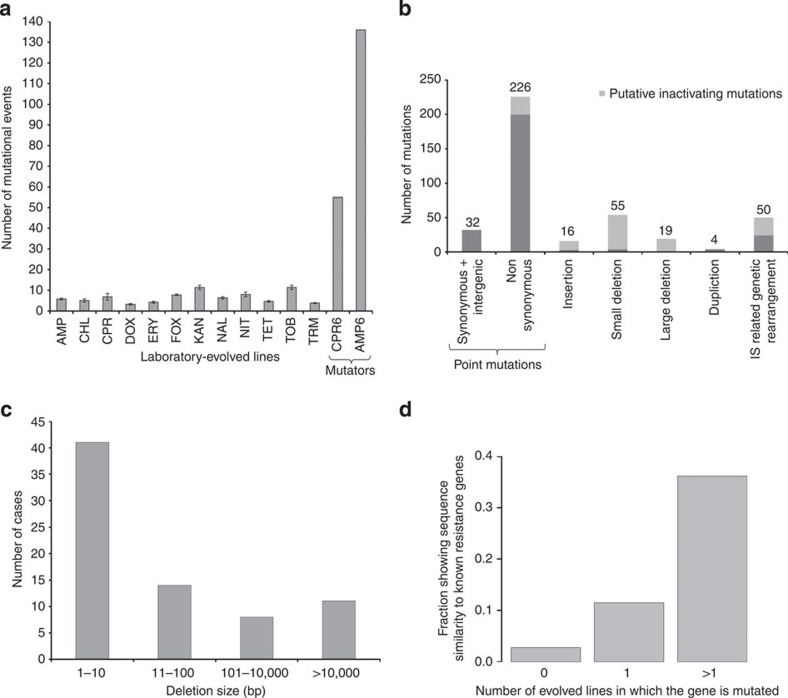
Mutations identified in independently evolved lines. Distribution of mutational events according to antibiotic (**a**), type (**b**) and size of DNA deletions (**c**). Laboratory-evolved mutator lines have accumulated exceptionally large numbers of mutations. The total number of putative loss-of-function mutations among point mutations, insertions and small deletions is 27% (**b**). (**d**) Observed mutations and known antibiotic resistance genes. Genes mutated in evolved lines are more likely to show significant sequence similarity to known antibiotic resistance genes^16^ than non-mutated ones (28 out of 143 versus 120 out of 4,358, *P*<10^−14^, Fisher’s exact test). Furthermore, genes showing sequence similarity to known resistance genes are enriched among genes mutated in multiple lines compared with those mutated in a single line (17 out of 47 versus 11 out of 96, *P*<0.005, Fisher's exact test). We identified genes showing significant sequence similarity to a set of genes curated in the Comprehensive Antibiotic Resistance Database^16^ using BLASTP search. In brief, we used the standalone NCBI BLASTP+ tool to identify *E. coli* genes that show highly significant similarity to any of the curated resistance or target genes (a conservative *E*-value cutoff of 10^−30^ was applied).

To gain insights into the underlying molecular mechanisms, we selected 63 independently evolved lines from the final day of experiments (5–6 lines per antibiotic). These lines were subjected to whole-genome sequencing using the Applied Biosystems SOLiD platform. We implemented an established computational pipeline to identify mutations relative to the ancestral genome (see Methods). To ensure that our pipeline correctly identified true mutations, a set of randomly chosen structural variants, such as point mutations, deletions, inversions and duplications, were validated by independent methods, that is, Sanger sequencing and qPCR. Altogether 16 validations were performed and the results are in complete agreement with the whole-genome sequencing data (Supplementary Table 2). Mutator bacterial populations have frequently been associated with decreased antibiotic susceptibility in clinics^13^,^14^ and laboratory evolution^15^. In agreement with this general trend, two evolved lines exerted elevated genomic mutation rates due to mutations in methyl-directed mismatch repair and in the DNA proof-reading subunit of DNA polymerase III (Supplementary Fig. 1). As a consequence, these lines accumulated exceptionally large numbers of mutations (synonymous and non-synonymous alike), many of which were unlikely to be functionally relevant (Fig. 2a and Supplementary Data 3). Therefore, these lines were excluded from all further analyses.

For the remaining 61 lines, we identified 402 independent mutational events (SNPs, insertions, small and large genomic rearrangements). On average, we detected 4.2 point mutations, 1.2 deletions, 0.26 insertions and 0.07 duplications per clone (Fig. 2a,b). Deletions were generally short (1–100 bp), with 19 major exceptions that span over 0.3–58 kb and eliminated 1–61 genes (Fig. 2c and Supplementary Data 3). Insertion sequences (IS) initiated large-scale genomic rearrangements (inversion, transposition or duplication) and were observed in 59% of the laboratory-evolved lines (Supplementary Data 3).

Several lines of evidence indicate that the accumulation of the mutations in protein-coding regions was largely driven by selection towards increased resistance. First, 87% of point mutations were non-synonymous. Second, at least 19% of the mutated genes showed significant sequence similarity to known antibiotic resistance genes^16^ (Fig. 2d and Supplementary Data 4), and several observed substitutions were previously found in natural or clinical isolates (Supplementary Data 5).

Consistent with prior studies^17^, antibiotic resistance generally conferred a measurable fitness cost: at least 41% of the laboratory-evolved lines showed a significantly reduced growth in antibiotic-free medium compared to the wild-type. As expected, lines with especially low fitness values in antibiotic-free medium have accumulated large numbers of mutations, including deletions of large genomic segments (Supplementary Fig. 2).

### Loss-of-function mutations are prevalent

Over 27% of the observed point mutations, small deletions and insertions generated in-frame stop codons, frameshifts or disruption of the start codon. These mutations were most likely to yield proteins with compromised or no activities (Fig. 2b and Supplementary Data 3). This figure is significantly higher than that observed in a previous large-scale laboratory evolutionary experiment towards high temperature^18^ (90 out of 329 versus 145 out of 1,030, Fisher’s exact test, *P*=1.017 × 10^−7^). Furthermore, the frequency of nonsense mutations among point mutations is three-fold higher than expected, based on the spontaneous mutation rate inferred from whole-genome sequencing of mutation-accumulation lines^19^ (26 out of 258 versus 8 out of 233, Fisher’s exact test, *P*<0.005). This result indicates widespread positive selection on inactivating mutations in our data set. Comparison with chemogenomic data^20^ indicated that inactivation of the corresponding genes tends to reduce antibiotic susceptibility compared with that of all other genes in the *E. coli* genome (14 out of 43 versus 321 out of 3,933, Fisher’s exact test, *P*<10^−5^). In many cases, the null mutations enhanced resistance to multiple drugs (Supplementary Data 6). For example, loss-of-function mutations occurred repeatedly in transcriptional repressors of antibiotic stress response (for example, *acrR*, *marR* and *mprA*). Similarly, IS-related inversions and transpositions frequently disrupted genes with known influence on antibiotic susceptibility. For instance, loss-of-function mutations in the NADPH nitroreductase genes (*nfsA* and *nfsB*) cause resistance to nitrofurantoin and related agents^21^. These genes were disrupted four times independently in nitrofurantoin-evolved lines (for other examples, see Supplementary Data 3).

### Evidence for parallel evolution

**Figure 3:**
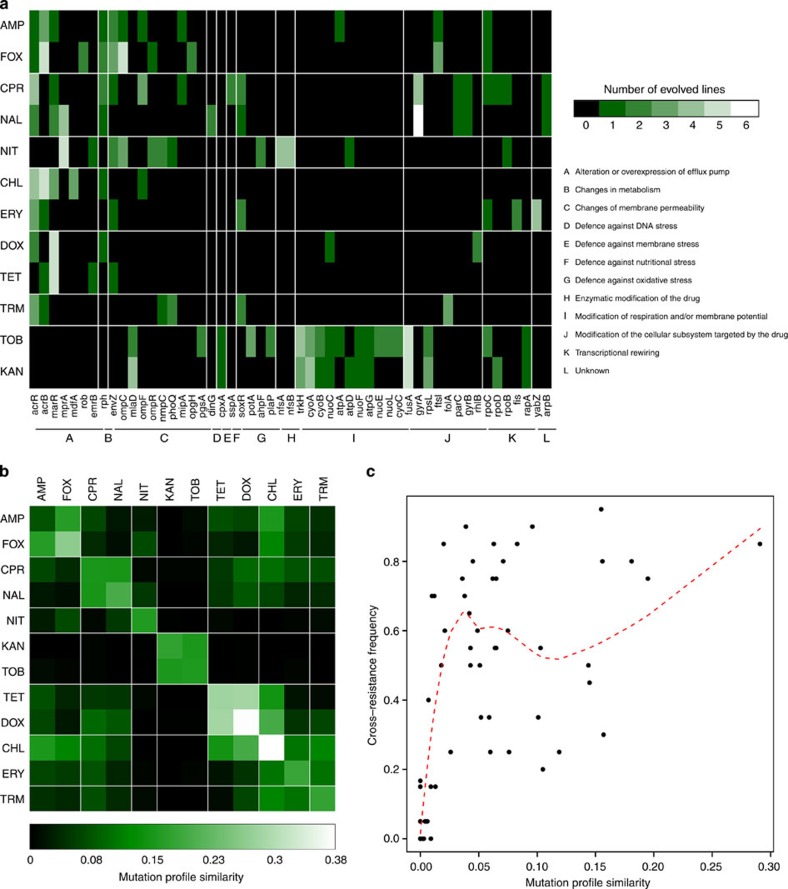
Parallel evolution and cross-resistance. (**a**) Mutational profiles of the 12 antibiotic selection regimes. Only those genes are shown that mutated in two or more of the 61 sequenced non-mutator laboratory-evolved lines. Mutations in promoters of multi-genic operons were associated with all genes encoded by the operon. The colour code indicates the number of cases when the same gene was independently mutated in different lines evolved under the same antibiotic pressure. (**b**) Heatmap of the average mutation profile similarity of two strains adapted to different (off-diagonal elements) and identical (diagonal elements) antibiotics. Mutation profile similarity between each pair of evolved lines was estimated by the Jaccard’s coefficient between their sets of mutated genes. Note that the map is symmetric. (**c**) Very-low average mutation profile similarities between strains adapted to different antibiotics are associated with low cross-resistance frequencies between antibiotic pairs. Mutation profile similarity was calculated as in **b**. Antibiotic pairs with mutation profile similarities <0.01 show significantly lower cross-resistance frequencies than the rest of the pairs (*P*<10^−10^, *N*=66, Wilcoxon rank-sum test), even when aminoglycosides are excluded (*P*<0.005, *N*=45). Dashed red curve indicates a smooth curve fitted by Loess regression^56^ (using the local polynomial regression fitting function of R).

A strong pattern of parallel evolution emerged at the level of amino-acid sites, genes and functional modules. Eight per cent of the point mutations were shared by at least two lines, and some were shared extensively (Supplementary Data 3). For example, a specific mutation (Val1127Gly) in a subunit (*acrB*) of the AcrAB/TolC efflux system was shared by four lines adapted to three different antibiotics (CHL, AMP and FOX). A total 35% of the affected genes were mutated repeatedly (Fig. 3a and Supplementary Table 3). These repeatedly mutated genes were especially likely to show significant sequence similarity to known antibiotic resistance genes^16^ (Fig. 2d and Supplementary Data 4), and some were frequently found in clinical multidrug-resistant strains^22^,^23^,^24^,^25^,^26^,^27^,^28^. Similarly, 2% of the observed small deletion events (1–82 bp) and 75% of the large deletion events (0.3–58 kbp) were at identical or nearly identical positions in two or more lines (Supplementary Data 3). These large deletions were generally flanked by homologous IS elements, suggesting that these deletions were mediated by recombination events between IS elements (Supplementary Data 3).

The above figures are all the more surprising as 66% of all parallel mutated genes occurred in lines adapted to different antibiotics. These results indicate that despite substantial differences in antibiotic treatments, the ultimate targets of antibiotic selection are overlapping functional modules. To investigate this issue further, we grouped 88% of the mutations into several major resistance mechanisms based on literature data (Table 2). The following major conclusions can be drawn.

First, mutations in the subsystem targeted by the antibiotic were only found in 49% of the laboratory-evolved lines. The absence of target mutations in the remaining lines may reflect unusually high associated fitness costs^5^, rarity of appropriate mutations and/or the efficiency of alternative resistance mechanisms (such as modification of efflux mechanisms, see Table 2). Mutations putatively affecting enzymatic modification of the antibiotic were observed in nitrofurantoin-adapted lines only (Table 2).

Second, genes involved in membrane transport, porin biosynthesis and membrane permeability were repeatedly mutated (Table 2), especially in lines adapted to protein synthesis inhibitors and quinolones. In sharp contrast, such mutations were conspicuously absent in aminoglycoside-resistant populations (Table 2, see also ref. 7).

Third, transcriptional regulatory genes were highly enriched in the set of accumulated mutations (Table 2). Many of them belong to specific two-component regulatory systems, and mediate general cellular defence against stressful conditions. These conditions include osmotic (OmpR/EnvZ, AcrR), acidic (PhoQ), metal (ComR), membrane (CpxR), antibiotic and oxidative stresses (MarA/SoxS/Rob regulon). Consistent with their roles in antibiotic tolerance^29^, global transcriptional regulatory proteins (RpoC, Crp and Fis) were also occasionally mutated.

Fourth and more generally, nutrient and oxidative stress response pathways were mutated in response to several different antibiotic stresses (Table 2). Consistent with prior studies on antibiotic tolerance^30^,^31^, central components of the stringent response (SpoT and SspA) were occasional targets of selection. Antioxidant stress response (SoxR and AhpF)^32^ and production of antioxidant molecules^33^, such as putrescine and spermidine, were also selected under antibiotic selection (Supplementary Data 3). In response to DNA-damaging antibiotic stress, populations mutated members of the SOS regulon (*dinB*, *yafO* and *yafP*) and cryptic prophages (cryptic prophage CP4-44). Indeed, prophages provide enhanced survival of the bacterial host in times of antibiotic stress^34^.

### Cross-resistance and parallel molecular evolution are linked

Despite differences in antibiotic selection pressure, parallel evolution was prevalent at multiple levels. This pattern is very unlikely to reflect adaptation unrelated to antibiotic treatment, as such parallel mutations generally incurred a fitness cost in antibiotic-free medium (see below). We hypothesized that parallel evolving mutations have an important contribution to the observed cross-resistance interactions. To investigate this issue, we calculated the average fraction of mutated genes shared by two strains for each pair of antibiotics (Fig. 3b).

Adaptation to certain antibiotics proceeds through diverse combinations of mutations (for example, on average, pairs of nitrofurantoin-adapted strains show 16.5% overlap in their sets of mutated genes), while the number of evolutionary trajectories appear to be more limited in other cases (for example, the same figure for chloramphenicol is 38%). Antibiotic pairs that have an especially low overlap in the corresponding sets of their mutated genes rarely displayed cross-resistance (Fig. 3c; *P*<10^−10^, *N*=66, Wilcoxon rank-sum test when pairs with a mutation profile similarity of <0.01 were compared with the rest). This pattern can be largely, but not exclusively, attributed to aminoglycosides: the sets of genes mutated under aminoglycoside selection pressure displayed practically no overlap with those detected in other laboratory-evolved lines (Fig. 3b), and cross-resistance was also absent. However, the association between low mutational overlap and scarcity of cross-resistance remains even when aminoglycosides are excluded from the analysis (*P*<0.005, *N*=45, Wilcoxon rank-sum test).

To investigate the role of parallel evolving mutations in cross-resistance further, we selected seven genes for further characterization, all of which were mutated in multiple laboratory-evolved lines and cover a wide range of molecular functions. The selected mutations were inserted individually into wild-type *E. coli*. The mutations generally conferred mild, but significant declines in susceptibilities to several antibiotics (Table 3). For example, a mutation in PhoQ, a member of the two-component regulatory system involved in acid and low Mg^2+^ stress tolerance^35^, increased resistance both to cell wall inhibitors and to the folic acid inhibitor trimethoprim. Beyond their beneficial effects, the selected mutations frequently had significant fitness costs in antibiotic-free medium (ref. 17) and enhanced sensitivity to certain antimicrobial agents (Table 3). The cross-resistance patterns conferred by individual mutations and the corresponding laboratory-evolved lines showed 62% overlap (45% would be expected by chance, randomization test, *P*=0.002, *N*=144 and Supplementary Data 7).

### Antibiotic features and cross-resistance patterns

By compiling a data set on the chemical and functional properties of antibiotics, we next examined the extent to which similarities in individual antibiotic properties shape the distribution of cross-resistance interactions in the network. One might argue that cross-resistance occurs mainly between antibiotics that target the same cellular subsystems. However, target mutations were present in less than half of the evolved lines and 88% of the cross-resistance interactions occurred between antibiotics with different cellular targets.

**Figure 4:**
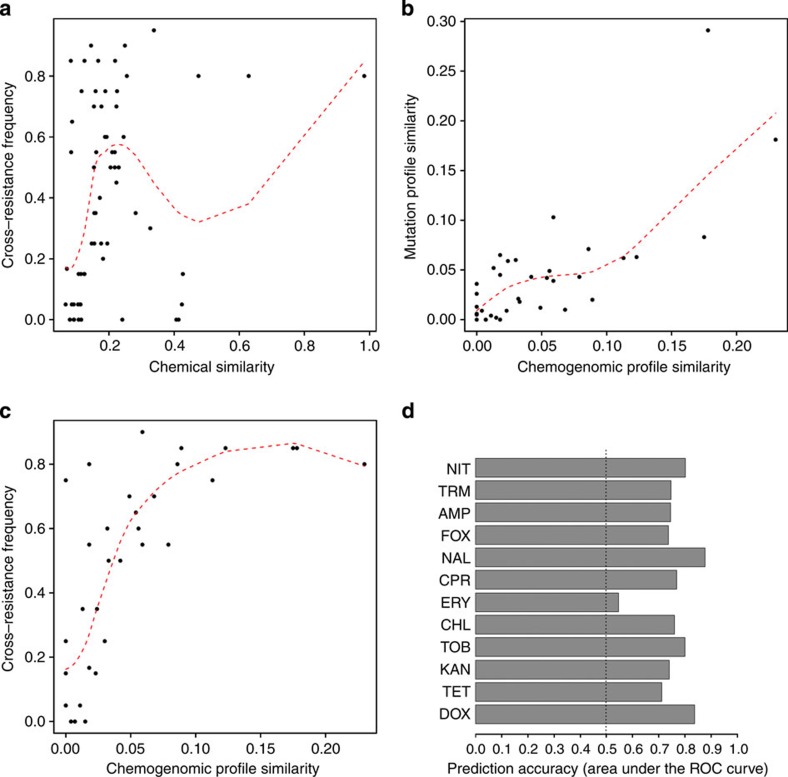
Antibiotic properties and cross-resistance. (**a**) Weak association between chemical structural similarity between antibiotic pairs and cross-resistance frequency (Spearman’s *ρ*=0.40, *P*<10^−3^, *N*=66), which disappears when aminoglycosides are excluded (*ρ*=0.21, *P*=0.18, *N*=45). Structural similarity between antibiotics was estimated by the Tanimoto similarity of their molecular fingerprints. (**b**) Correlation between chemogenomic profile similarity and overlap in the set of accumulated mutations during laboratory evolution (Spearman’s *ρ*=0.67, *P*<10^−5^, *N*=36). (**c**) Antibiotic pairs that frequently display cross-resistance interactions show relatively high overlap in their chemogenomic profiles (Spearman’s *ρ*=0.77, *P*<10^−7^, *N*=36). Dashed red curves on scatterplots A–C indicate smooth curves fitted by Loess regression^56^. (**d**) Predicting antibiotic resistance phenotypes from genome sequences. Prediction performance for each antibiotic based on the set of accumulated mutations was measured by the area under the receiver operating characteristic (ROC) curve (AUC). This gives an overall measure of accuracy by taking into account both true positive and false positive rates across all possible cutoffs of the prediction score. Random prediction gives an AUC of 0.5. Variation in resistance among evolved strains can be predicted with 55–88% (76% average) accuracy, depending on the antibiotic studied. Special care was taken to avoid circularity in the predictions.

Relatedness of chemical structures (as captured by chemical fingerprint similarities as measured by the Tanimoto coefficient^36^) emerges as a weak predictor of antibiotic cross-resistance (Spearman’s *ρ*=0.4, *P*<10^−3^, *N*=66, Fig. 4a). Furthermore, this marginal effect is entirely attributable to aminoglycosides, which have low chemical similarity with other antibiotics and rarely show cross-resistance interactions with them (Spearman’s *ρ*=0.21, *P*=0.17, *N*=45 when excluding aminoglycosides).

Last, the intrinsic resistome (that is, the set of genes that influence antibiotic sensitivity) provides an unbiased description of antibiotic action^37^. We, therefore, asked how the overlap in the intrinsic resistome shapes the distribution of cross-resistance interactions. Our molecular and phenotypic results were integrated with data from a previous chemogenomic screen^20^. That study exposed a nearly complete mutagenized *E. coli* library to each of 17 antibiotics and determined the fitness contribution of individual genes. Using this data set, we calculated the sets of genes that influence susceptibility for each antibiotic used in our study (chemogenomic profile). Strikingly, antibiotic pairs that showed substantial overlap in their chemogenomic profiles also accumulated similar sets of mutations during the course of laboratory evolution (Spearman’s *ρ*=0.67, *P*<10^−5^, *N*=36; Fig. 4b), and frequently displayed cross-resistance interactions (Spearman’s *ρ*=0.78, *P*<10^−7^, *N*=36; Fig. 4c). Importantly, these results remained when excluding antibiotic pairs targeting the same subsystem (Spearman’s *ρ*=0.59, *P*<10^−3^, *N*=33 and Spearman’s *ρ*=0.73, *P*<10^−5^, *N*=33, respectively) or those involving aminoglycosides (Spearman’s *ρ*=0.57, *P*<0.005, *N*=28 and Spearman’s *ρ*=0.75, *P*<10^−5^, *N*=28, respectively).

### Mutational analysis captures antibiotic resistance profile

Our data indicate that the molecular mechanisms of antibiotic resistance evolve in a repeatable manner. This raises the question whether it is possible to predict antibiotic resistance phenotypes from the genome sequences of the laboratory-evolved lines. We employed a simple procedure that uses gene sets derived from our sequenced evolved lines to predict differences in resistance phenotypes among individual strains. Briefly, for each antibiotic, we compiled the complete list of genes that were mutated at least once in lines evolved under the given antibiotic selection pressure. This gene–antibiotic association set was compared with the set of genes mutated in each strain with known antibiotic resistance profile, resulting in a set of 12 scores measuring the likelihood of resistance of the evolved line against the complete panel of 12 antibiotics. The above procedure was repeated for each of our 61 sequenced evolved lines in turn. To quantify the agreement between this simple prediction score against experimentally determined resistance profiles (that is, increased resistance compared with wild-type), we used a combined measure of sensitivity (true positive rate) and specificity (true negative rate)^38^. In particular, we measured how accurately our prediction procedure separates resistance and sensitivity to a given antibiotic when averaged across all 61 evolved lines. The analyses demonstrated that variation in antibiotic resistance among evolved strains can be predicted with an average 76% accuracy, while only 50% would be expected by chance (Fig. 4d and Supplementary Fig. 3). For example, the method is able to discriminate doxycycline-resistant and sensitive strains with 84% accuracy. We emphasize that our attempt to predict resistance profiles is preliminary and future works should investigate whether incorporation of more antibiotics, a greater diversity of genomes or usage of more refined prediction algorithms could improve prediction success.

## Discussion

By combining experimental evolution, genome sequencing and functional analyses, this work charted a map of cross-resistance interactions between antibiotics in *E. coli*, and explored, on a genome-wide scale, the mechanisms driving these evolutionary patterns. The following general conclusions can be drawn from our study.

First, our work indicates that the progressive accumulation of spontaneous mutations under antibiotic selection simultaneously changes the organism’s sensitivity to many other antimicrobial agents (Fig. 1a). It also revealed differences in the efficacy by which different antibiotics can inhibit growth of resistant bacterial populations or select for the emergence of multidrug-resistant strains (Fig. 1c). Cross-resistance between two antibiotics was largely independent of whether they show synergistic effects in combination^12^,^39^. Thus, the networks based on evolutionary and physiological antibiotic interactions are generally governed by distinct mechanisms. As both synergism and cross-resistance interactions between antibiotic pairs can potentially influence long-term evolutionary pathways^4^, combination of these two maps could be especially informative for future development of novel antimicrobial strategies.

Second, a strong signature of parallel evolution emerged across populations adapted to different antibiotics (Table 2), although the molecular mechanisms underlying antibiotic resistance and cross-resistance were diverse. Our work identified several genes where the observed mutations delivered resistance to multiple antimicrobial agents (Supplementary Table 3). In several instances (*phoQ*, *envZ*, *soxR* and *trkH*), the potential roles of these genes in multidrug resistance are yet to be investigated in the clinic. Unexpectedly, even a mutation in the molecular target of the antibiotic can alter sensitivity to multiple, unrelated antibiotics. Laboratory-evolved fluoroquinolone resistant lines frequently exhibited a specific mutation in the target topoisomerase gene (*gyrA*: A87G). This single mutation influenced sensitivity to several non-quinolone drugs (Table 3), probably through altering patterns of supercoiling and hence global expression of stress response pathways^40^. Strikingly, in several instances, individual mutations simultaneously enhanced sensitivity to other drugs (Table 3), indicating that negative trade-offs (collateral sensitivity interactions) are prevalent during antibiotic selection^6^,^7^,^8^,^41^,^42^,^43^. More generally, the presence of parallel mutations allowed us to predict the resistance profiles of evolved lines from their genome sequence based on catalogues of genes mutated under different antibiotic selection pressures.

Third, as high as 27% of the observed mutations generated proteins with compromised or no activities (Fig. 2b). While potential roles of loss-of-function mutations during antibiotic evolution have been suggested^22^,^44^,^45^, our work provides the first estimate on the relative importance of this mutational class. Given their high rates and potential beneficial effects, loss-of-function mutations could play an important role during the early stage of resistance evolution (see also ref. 46).

Fourth, chemogenomic profile similarity between antibiotics emerges as the most significant determinant of cross-resistance (Fig. 4c). Thus, beyond their pivotal role in elucidating the mechanisms of drug actions^47^, systematic chemogenomic studies could also be used in the future to infer general trends of resistance evolution.

Taken together, our analyses indicate that resistance evolution is governed by highly pleiotropic mutations in a relatively limited set of functional modules. The prevalence of mutations with pleiotropic effects indicates that the phenomenon of cross-protection may be more general and extend to other stressful conditions unrelated to antibiotic pressure^48^. Indeed, genes mutated in our study were enriched in the set of *E. coli* genes that influence sensitivity to toxic metal (for example, copper and nickel) and detergent exposure (Supplementary Table 4). Given the documented associations between levels of metal contamination and specific patterns of antibiotic tolerance in nature^49^, future evolutionary studies should investigate how frequently metal and antibiotic resistance are co-selected in the laboratory. It will also be important to establish to what extent cross-resistance interactions remain conserved across (pathogenic) species or depend on the introduction of novel genes by horizontal transfer. As most laboratory-evolved lines displayed relatively low fitness in antibiotic-free medium, it will also be important to establish the extent to which adaptation through compensatory mutations can mitigate the costs of resistance.

More generally, understanding the fitness consequences of genetic adaptations to different environments remains an important challenge for evolutionary biology^1^. Thanks to the recent availability of the necessary computational tools and experimental techniques, it has become possible to predict certain aspects of genomic evolution^50^. Integrating experimental evolution, systems biology and genomics in a framework similar to that presented in this paper could result in the inference of general rules underlying the evolutionary trade-offs observed in nature.

## Methods

### Laboratory evolutionary experiment

Details of the laboratory evolution experiments have been described elsewhere^7^. Briefly, populations of *E. coli* K12 were grown in MS-minimal medium supplemented with glucose, casamino acids and 1 of 12 possible antibiotics. Parallel cultures were propagated in 96-well microtiter plates. Bacterial cells were transferred every 24 h by inoculating ~1% of the culture to 100 μl fresh medium. Starting with a subinhibitory (IC50) antibiotic concentration, antibiotic dosage was increased gradually (1.5 times the previous dosage) at every fourth transfer. We propagated 96 independent populations in the presence of each antibiotic up to ~336 generations. As expected, population sizes differed significantly across treatments and antibiotic dosages, reflecting independent evolutionary trajectories. For each antibiotic, the experiment halted at the last antibiotic dosage that permitted growth of at least 10 out of 96 parallel evolving populations (criteria was defined as the failure to obtain growth OD 600<0.05) or when the antibiotic concentration had reached its upper solubility limit (Supplementary Table 5). For each antibiotic, 10 populations with the highest final cell densities were selected for further analysis, resulting in 120 parallel evolved lines. We also established 10 parallel populations growing in an environment devoid of antibiotics for the same number of transfers, referred to as adapted control lines.

### Measurement of antibiotic susceptibilities

Given a panel of resistant strains, our next goal was to detect changes in their sensitivity towards other antimicrobial agents. We developed a highly sensitive high-throughput screening and a robust statistical methodology^7^. Briefly, we tested the susceptibility of each evolved and control lines against the entire set of antibiotics by measuring their growth in liquid cultures at sublethal doses of antibiotics. Bacterial growth was monitored by measuring optical density (OD 600) of the liquid cultures at a single time point after 14 h of growth^7^. Prior experiments demonstrated that a single reading of optical density shows very strong linear correlation with the area under the growth curve^7^.

To identify statistically significant cross-resistance interactions, we tested whether each evolved line showed a significant growth difference from the set of 10 control lines. To do this, for each evolved line, we calculated the median value of the four technical replicates and compared it with the distribution of the median growth values of the four technical replicates of the 10 control lines using a Z-test. This yielded a matrix of evolutionary interactions between evolved strains and antibiotics (Supplementary Data 1). Where multiple independent experimental runs were available, we used Fisher’s method to aggregate *P*-values. All statistical analyses were carried out in Matlab. The results were confirmed by *E*-test assays, using standard protocols. Finally we calculated the the frequency of cross-resistance (FCR) for each antibiotic pair as follows: FCR=(NA→B+NB→A)/(NA+NB), where NA→B and NB→A are the number of populations adapted to antibiotic A showing enhanced resistance to B, and *vice versa*. NA and NB are the total number of populations adapted to antibiotic A and B, respectively.

### Chemical and chemogenomic profile similarities

Chemical similarities of antibiotics were computed using an R implementation of the cheminformatics library CDK (Chemistry Development Kit)^51^. Chemical relatedness was captured by chemical fingerprint similarity as measured by the standard Tanimoto coefficient^52^. Chemogenomic similarity was calculated as pair-wise Jaccard similarity coefficient between sets of genes that influence antibiotic susceptibility based on a published chemogenomic screen^20^. This chemogenomic screen covered 9 of the 12 antibiotics employed in our study, and as it relied on a highly sensitive competition assay, it was particularly useful to identify genes whose inactivation increased antibiotic tolerance. MIC and dose response curve measurements were performed as described previously^7^.

### Whole-genome sequencing

The ancestral and 63 selected evolved strains were subjected to next-generation sequencing to identify mutations. Genomic DNA (gDNA) was extracted from selected *E. coli* strains (SIGMA GenElute Bacterial Genomic DNA kit) and the subsequent library preparation was performed using the 5500 SOLiD Fragment Library Core Kit (Life Technologies; LT). Briefly, 3 μg of purified bacterial gDNA was fragmented by Covaris S2 System to 100–250 bp. The fragmented DNA was end-repaired and ligated to P1 and P2 adaptors, which provide the primary sequences for both amplification and sequencing of the sample library fragments; the P2 adaptor contains a 10-bp barcode sequence that provided the basis for multiplex sequencing (5500 SOLiD Fragment Library Barcode Adaptors; LT). The templates were size-selected using Agencourt AMPure XP system (Beckman Coulter), nick-translated using Platinum PCR Amplification Mix and the template library was quantified by qPCR using SOLiD Library TaqMan Quantitation Kit (LT). The templates were clonally amplified by emulsion PCR (ePCR) with P1 primer covalently attached to the bead surface. Emulsions were broken with butanol and ePCR beads enriched for template-positive beads by hybridization with P2-coated capture beads. Template-enriched beads were extended at the 3′ end in the presence of terminal transferase and 3' bead linker. Beads with clonally amplified DNA were then deposited onto a SOLiD Flowchip and the slide was loaded into a SOLiD 5500xl System (LT) and the 50-base sequences were obtained according to the manufacturer’s protocol.

### Bioinformatic analysis of genome sequences

The obtained sequences from each strain were first trimmed in order to filter out low-quality reads that were shorter than 50 bp. The remaining high quality sequences from each strain were then aligned to the *E. coli* K-12 substr. MG1655 chromosome (GenBank Accession No. NC000913; Version NC_000913.2 GI:49175990) in colour space using Genomics Workbench 6.5 (CLC Bio). Within a single read, the maximum gap and mismatch count was set to two and the similarity fraction was set to 0.8. Two mappings were performed for each strain which differed in setting the length fraction to 0.5 for relaxed or 0.6 for stringent analysis. Minimum coverage of ≥51-fold and ≥44-fold was accomplished for each strain when using relaxed or stringent parameters, respectively. A minimum of six reads were required to call a point mutation or short indel (<15 bp) upon relaxed analysis; in contrast, 20 reads were required to call a structural variation (SV; for example, inversion, duplication, replacement, translocation) upon stringent analysis.

For quality-based variant detection we used an approach based on the Neighbourhood Quality Standard algorithm that is implemented in Genomics Workbench. Relaxed alignment was used to identify point mutations or short indels; the minimum variant frequency was set to 50%. Variants identified in the ancestral genome were excluded from further analyses. All remaining potential variants were manually checked with a visual output in order to exclude false variant calls due to insufficient mapping accuracy.

The soft-clipped, unaligned ends of the sequence reads were used to map SVs and long indels. For this, stringent alignment was used and the resulting self-mapped, cross-mapped, multiple, close and paired breakpoints (for details see http://www.clcsupport.com/clcgenomicsworkbench/current/) were identified and manually checked; indels and SVs identified in the ancestral genome were again excluded. All identified breakpoints were validated by re-mapping: consensus sequence resulting from large indel or SV was extracted, re-mapping was performed using stringent setup and the breakpoint considered valid if perfectly matching sequence tags overlapped the breakpoint.

### Validation of whole-genome sequencing data

Several structural variants were randomly chosen and validated by either PCR followed by Sanger sequencing (for example, point mutations, deletions and inversions) or by quantitative PCR (for example, duplications). For this latter, DNA levels were determined using StepOne Plus Real-Time PCR system (LT). Reactions were performed by using Power SybrGreen Master Mix (LT); the primer sequences are available on request. All of the measurements were performed in duplicates; the ratio of each amplicon relative to the normalizing control was calculated using the 2^−ΔΔCT^ method.

### Allele replacements

Allele replacements were constructed by a suicide plasmid-based method. Standard steps and plasmids (pST76-A, pSTKST) of the procedure were described previously^53^. In brief, an ~800-bp long targeting DNA fragment carrying the desired point mutation in the middle was synthesized by PCR, then cloned into a thermosensitive suicide plasmid. The plasmid construct was then transformed into the cell, where it was able to integrate into the chromosome by way of a single crossover between the mutant allele and the corresponding chromosomal region. The desired cointegrates were selected by the antibiotic resistence carried on the plasmid at a nonpermissive temperature for plasmid replication. Next, the pSTKST helper plasmid was transformed, then induced within the cells, resulting in the expression of the I-SceI meganuclease enzyme, which cleaves the chromosome at the 18-bp recognition site present on the integrated plasmid. The resulting chromosomal gap is repaired by way of RecA-mediated intramolecular recombination between the homologous segments in the vicinity of the broken ends. The recombinational repair results in either a reversion to the wild-type chromosome, or in a markerless allele replacement, which can be distinguished by sequencing of the given chromosomal region. For all primers, see Supplementary Table 6.

As other methods failed, the oligonucleotide-mediated λ Red recombination was used to generate the *gyrA* variant S83→L and D87→G in *E. coli* BW25113. The applied wild-type strain contained the pBADαβγ λ Red expression plasmid for inducible λ Red recombinase production. Oligonucleotides for allelic replacement were designed according to standard guidelines^54^. Briefly, oligos applied for allelic replacement have complementary sequences to the replicating lagging strand and have minimized secondary structure (less than −12 kcal mol^−1^). Additionally, each oligo contained two subsequent phosphorothioate linkages at both 5′ and 3′ termini for endogenous nuclease evasion. Oligos were ordered with standard purification and desalting from Integrated DNA Technologies (IDT). To perform allelic replacement, cells were grown in 10 ml Luria Bertani (LB) broth, supplemented with 100 μg ml^−1^ ampicillin, from overnight starter culture at 37 °C, 250 r.p.m. to OD_550_ 0.5–0.7. Expression of λ Red proteins were induced by the addition of L-Arabinose at 0.2% concentration for 30 min. For recombination, cells were pelleted (3,800 r.p.m. for 7 min) and washed twice in ice-cold dH_2_O, resuspended in 160 μl dH_2_O. 40 μl cell suspension was electroporated with oligo GyrAS83L or GyrAD87G at 2.5 μM final concentration. Electroporated cells were allowed to recover in 10 ml LB at 37 °C overnight. Cells were plated on LB agar plates supplemented with 100 ng ml^−1^ ciprofloxacin. Clones with desired mutation were identified by sequencing target site in *gyrA* using GyrA2F and GyrA2R primers.

### Mutation rate measurements

Mutation rates of two laboratory-evolved lines (AMP6, CPR6) were measured by using rifampicin (Rif^s^ to Rif^r^) forward fluctuation test. The rifampicin minimum inhibitory concentration (MIC) for the two evolved lines does not differ from that of the control line. Overnight cultures (grown in LB broth, on 30 °C) were diluted to 10^4^ cells per ml and six parallel cultures per each line were started in glass tubes. After 24 h incubation at 30 °C, appropriate dilutions were plated to LB agar plates for CFU determination, and to LB agar plates containing 100 μg ml^−1^ rifampicin for detection of rifampicin resistant mutants. Colonies were counted after 24 and 48 h, respectively. Mutation rates were calculated by using the MSS maximum-likelihood method^55^.

### Predicting antibiotic resistance phenotypes from genomic data

To predict antibiotic resistance phenotypes from genome sequences of the evolved lines, we employed a procedure that uses gene sets derived from our sequenced evolved lines to predict differences in resistance phenotypes among individual genomes. First, for each antibiotic, we compiled the list of genes that were mutated in at least one of our lines evolved under the given antibiotic selection pressure (for example, genes mutated in ampicillin-evolved lines for ampicillin). To avoid circularity in the predictions, these gene–antibiotic association lists were defined by leaving out the genome (G_x_) for which resistance prediction was attempted (that is, yielding slightly different association lists for each G_x_). Next, for each antibiotic, we counted the number of protein-coding genes that are both mutated in G_x_ and present in the gene–antibiotic association list of the given antibiotic. This procedure results in a set of 12 scores measuring the likelihood of resistance of evolved line G_x_ against our panel of 12 antibiotics. Finally, the above procedure was repeated for each of our 61 sequenced evolved lines in turn. To quantify the agreement between this simple prediction score against experimentally determined resistance profiles (that is, increased resistance compared to wild-type), we used a combined measure of sensitivity (true positive rate) and specificity (true negative rate)^38^. In particular, we measured how accurately our prediction procedure separates resistance and sensitivity to a given antibiotic when averaged across all 61 evolved lines. We note that not all gene–antibiotic association lists were equally informative in the prediction process as mutations occurring in aminoglycoside-evolved lines were especially relevant to discriminate between the presence and absence of resistance to a number of antibiotics (Supplementary Table 7). This is unsurprising given the distinct mutational profiles and resistance mechanisms of aminoglycoside-adapted lines.

## Author contributions

B.P. and C.P. conceived and supervised the project; V.L., I.N. and R.S. designed the experiments; V.L., I.N., R.S., B.C., Á.N., B.H., A.V., M.H., B.B. and O.M. performed the experiments; V.L., I.N., Á.G., R.B.-F., G.F., B.S., B.K., B.P. and C.P. developed data analysis procedures and interpreted the data; C.P. and P.B. wrote the manuscript with contributions from all other authors.

## Additional information

**Accession codes:** The raw sequences and assemblies have been deposited in NCBI Bioproject database under the accession code PRJNA248327 (accession SRP042209). The individual accession numbers for the 64 deposited samples are as follows: SRR1297006, SRR1297043, SRR1297049, SRR1297054 to SRR1297056, SRR1297060 to SRR1297064, SRR1297067, SRR1297069, SRR1297073, SRR1297077, SRR1297079, SRR1297081, SRR1297096, SRR1297101, SRR1297103 to SRR1297107, SRR1297109, SRR1297112, SRR1297114, SRR1297117, SRR1297123 to SRR1297126, SRR1297129, SRR1297132, SRR1297133, SRR1297135, SRR1297137, SRR1297139, SRR1297142, SRR1297144, SRR1297148, SRR1297150, SRR1297153, SRR1297155, SRR1297157, SRR1297159, SRR1297160, SRR1297163, SRR1297168 to SRR1297173 and SRR1297175 to SRR1297184.

**How to cite this article:** Lázár, V. *et al.* Genome-wide analysis captures the determinants of the antibiotic cross-resistance interaction network. *Nat. Commun.* 5:4352 doi: 10.1038/ncomms5352 (2014).

## Supplementary Material

Supplementary information Supplementary Figures 1-3, Supplementary Tables 1-7 and Supplementary References

Supplementary Data 1 Dataset of cross-resistance interactions

Supplementary Data 2 Validation of cross-resistance interactions.

Supplementary Data 3 Mutations identified in 63 laboratory evolved populations.

Supplementary Data 4 Sequence similarity of mutated genes to known antibiotic resistance or target genes.

Supplementary Data 5 Specific substitutions which were previously found in antibiotic resistant clinical isolates

Supplementary Data 6 Mutations generating in-frame stop codons, frameshifts or disrupting start codons.

Supplementary Data 7 Cross-resistance pattern of the evolved lines carrying the selected specific mutations.

## Figures and Tables

**Table 1:** Antibiotics employed and their modes of actions.

**Table 2:** Map of repeatedly mutating functional units across antibiotic treatments.

**Table 3:** Selected individual mutations and their sensitivity profiles across antibiotics.
