# Supplementary material for: Genome-wide analysis captures the determinants of the antibiotic cross-resistance interaction network
Source: Nat Commun. 2014 Jul 8;5:4352. doi: 10.1038/ncomms5352 (PMC4102323; doi:10.1038/ncomms5352)
Supplement: Supplementary information — Supplementary Figures 1-3, Supplementary Tables 1-7 and Supplementary References [file ncomms5352-s1.pdf]

Supplementary Figures

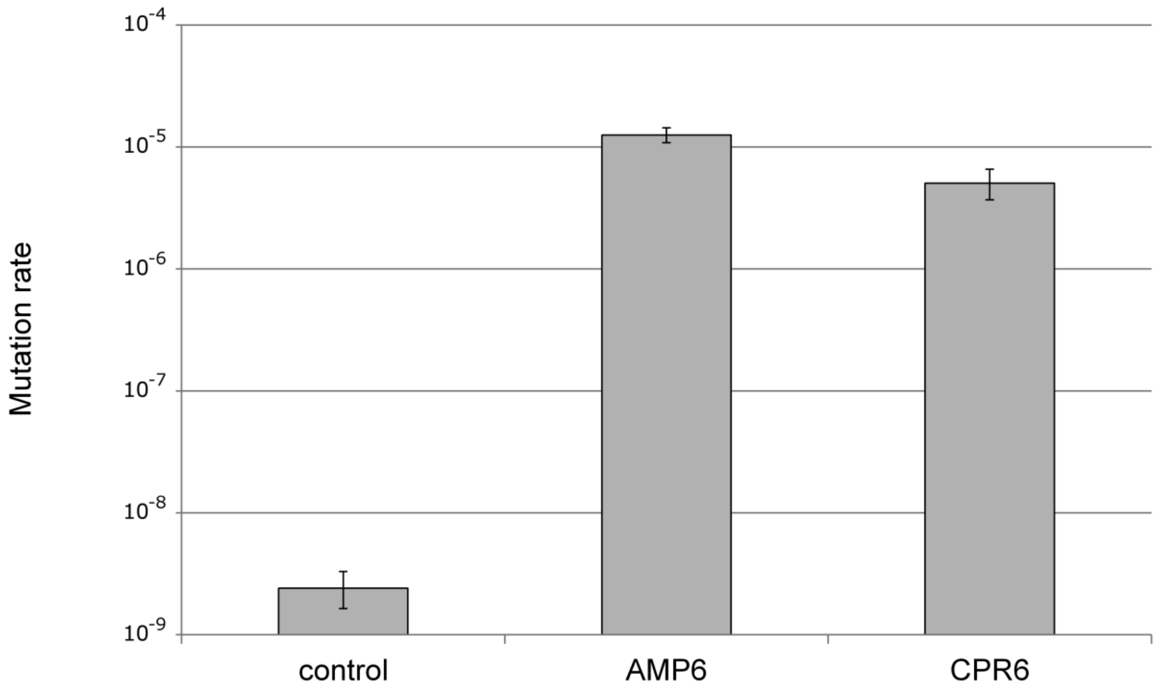

Supplementary Figure 1. Increased genomic mutation rates of two evolved lines. Mutation rate represents mutations/generation/cell. It was measured by using standard rifampicin fluctuation test and calculated with the MSS maximum-likelihood method (FALCOR web tool). Mutations in *dnaQ* (coding for the epsilon subunit of DNA polymerase III) and *mutL* (coding for a methyl-directed mismatch repair protein) genes, respectively, are partly responsible for the elevated mutation rates of AMP6 and CPR6 lines. Error bars represent 95% confidence intervals.

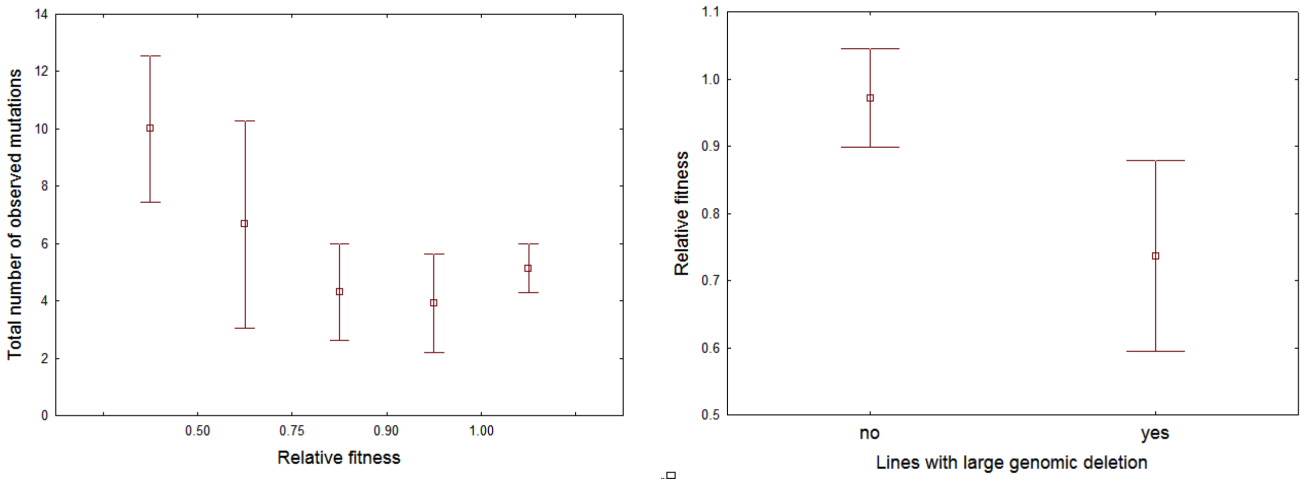

A **B**

Supplementary Figure 2 Mutations and the cost of evolved resistance in antibiotic-free medium

Fitness loss in antibiotic-free media (compared to that of wild type) was highly variable across laboratory evolved populations. Fitness was estimated by measuring optical density (OD 600) of the liquid cultures at a single time point by following previously developed protocols[1]. A) Lines with very low fitness in antibiotic-free medium have accumulated an especially high number of mutations (Kruskal Wallis test N=61, $P<10^{-4}$). B) Lines with deletions of large genomic segments showed especially low fitness values in antibiotic free-medium (N=61, t-test, P=0.0014). Error bars indicate 95% confidence intervals.

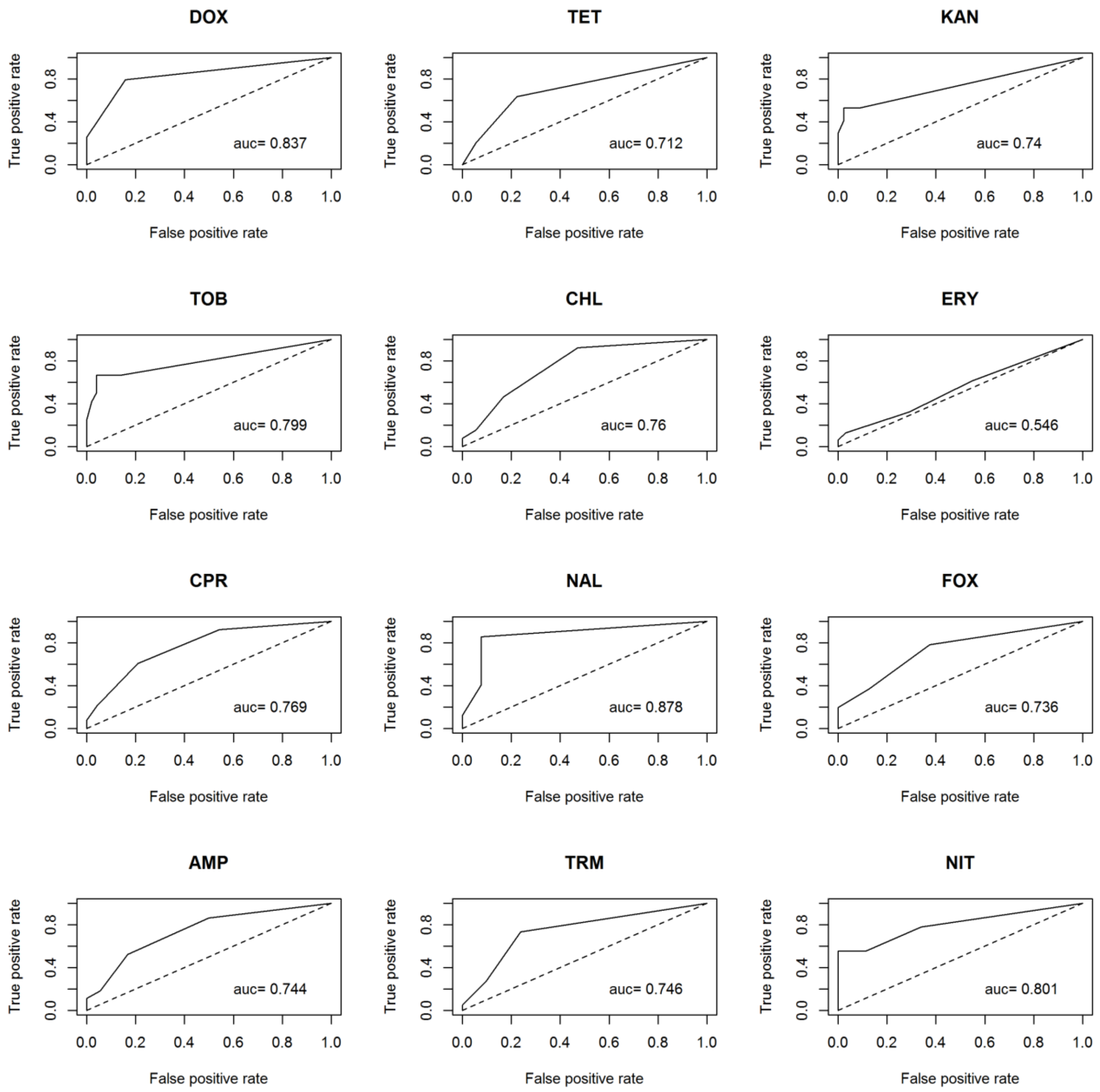

Supplementary Figure 3. Receiver Operator Characteristic (ROC) curves comparing the prediction performance for each antibiotic based on the set of accumulated mutations.

Prediction performance for each antibiotic based on the set of accumulated mutations was measured by the area under the receiver operating characteristic (ROC) curve (AUC). Random prediction gives an AUC of 0.5.

Supplementary Tables

Applied antibiotic	Number of transfers	Number of generations	MIC change
ampicillin	30	180 - 210	20-fold
ciprofloxacin	48	288 - 336	328-fold
tobramycin	45	270 - 315	146-fold
trimethoprim	36	216 - 252	43-fold
erythromycin	33	198 - 231	28-fold
tetracycline	39	234 - 273	65-fold
nitrofurantoin	30	180 - 210	20-fold
nalidixic	48	288 - 336	328-fold
cefoxitin	48	288 - 336	328-fold
kanamycin	45	270 - 315	146-fold
chloramphenicol	48	288 - 336	328-fold
doxycycline	30	180 - 210	20-fold

Supplementary Table 1 Estimated MIC changes following laboratory evolution towards gradually increased antibiotic dosage

line	duplication				deletion			inversion	
	sfmD	nfrA	mrdA	ykgH	pnp	ompF	sspA	genomic region	
	b0532	b0568	b0635	b0310	b3164	b0929	b3229	1906840.. ..2288136	1906830.. ..2065378
ERY5	+	+	+	n.r.	n.r.	n.r.	n.r.	n.r.	n.r.
FOX6	+	+	+	n.r.	n.r.	n.r.	n.r.	n.r.	n.r.
FOX8	+	+	+	n.r.	n.r.	n.r.	n.r.	n.r.	n.r.
TET7	+	n.r.	n.r.	n.r.	n.r.	n.r.	n.r.	n.r.	n.r.
TET1	n.r.	n.r.	n.r.	+	n.r.	n.r.	n.r.	n.r.	n.r.
NAL9	n.r.	n.r.	n.r.	n.r.	+	n.r.	n.r.	n.r.	n.r.
CPR9	n.r.	n.r.	n.r.	n.r.	n.r.	+	+	n.r.	n.r.
NIT4	n.r.	n.r.	n.r.	n.r.	n.r.	n.r.	n.r.	+	n.r.
TRM6	n.r.	n.r.	n.r.	n.r.	n.r.	n.r.	n.r.	n.r.	+

n.r.: not relevant

Supplementary Table 2. Validation of whole-genome sequencing data by Sanger sequencing or QPCR of 16 randomly chosen structural variants.

Gene	Function	Number of strains carrying mutation in this gene	Number of antibiotic classes towards which adaptation led to mutation in this gene
acrR	transcriptional repressor of the AcrAB efflux pump	20	5
marR	repressor of the *marRAB* operon, which controls an adaptation response	19	4
acrB	part of the AcrAB/TolC multidrug-efflux complex	16	4
envZ	regulates the expression of the major outer membrane porin genes	10	5
fusA	elongation factor	10	1
gyrA	DNA gyrase, subunit A	10	1
mprA	negatively regulates the transcription of multidrug resistance pumps	9	2
ompC	a major outer membrane porin	9	2
rph	Ribonuclease PH (RNase PH) activity	8	4
soxR	controls the transcription of the regulon involved in defense against redox-cycling drugs	7	3
cyoA	subunit II of the cytochrome bo terminal oxidase complex	7	1
trkH	potassium ion transporter	7	1
rpoC	RNA polymerase, β' subunit	6	4
mlaD	predicted substrate binding protein of the phospholipid ABC transporter	6	1
ompF	a major outer membrane porin	5	3
rpsL	component of the 30S subunit of the ribosome	5	1
ftsI	an essential cell division protein	4	1

Gene	Function	Number of strains carrying mutation in this gene	Number of antibiotic classes towards which adaptation led to mutation in this gene
nfsA	major oxygen-insensitive nitroreductase	4	1
nfsB	minor oxygen-insensitive nitroreductase	4	1
ycbZ	putative ATP-dependent protease	4	1
nfsA	nitroreductase	4	1
nfsB	nitroreductase	4	1
ompR	regulates the expression of the major outer membrane porin genes	3	2
phoQ	a transcriptional regulator that responds to Mg2+ limitation	3	2
rpoD	primary sigma factor during exponential growth	3	2
nmpC	outer membrane porin protein	3	2
cyoB	subunit I of the cytochrome bo terminal oxidase complex	3	1
folA	dihydrofolate reductase	3	1
potA	putrescine / spermidine ABC transporter	3	1
mdfA	multidrug efflux protein	3	1

Supplementary Table 3. Genes mutated repeatedly in laboratory evolved lines.

Environmental condition	Adjusted P-value
Copper stress (1 mM)	$2.6\ 10^{-6}$
Detergent / stress (SDS 0.5% + EDTA 0.5 mM)	$2.4\ 10^{-5}$
Temperature 20C	$2.4\ 10^{-5}$
Nickel stress (1 mM)	$2.7\ 10^{-5}$
Stress (EDTA 1 mM)	0.0004
Detergent (Benzalkonium 10 µg/ml)	0.001
Detergent (Bile 0.1%)	0.001
Temperature 16C	0.002
Ethanol stress (4 mM)	0.002
Acidic stress (pH 4)	0.003
Detergent (Cholate 1%)	0.004
Acetate as carbon source	0.004
Ethanol stress (6 mM)	0.004
Detergent (Benzalkonium 25 µg/ml)	0.006
Copper stress (4 mM)	0.006
Acidic stress (pH 5)	0.01
Detergent / stress (SDS 1% + EDTA 0.5 mM)	0.02
Detergent (SDS 0.5%)	0.03

Supplementary Table 4. Overlap between genes mutated in the antibiotic-evolved lines and those influencing sensitivity to other stress conditions.

Genes involved in modulating stress susceptibility were previously identified by growth profiling of a mutant library of *E. coli* under diverse environmental conditions[2]. Here, we considered abiotic stress conditions only and excluded antibiotic treatments. For each stress condition, we used a hypergeometric test to examine whether the list of genes either positively or negatively influencing sensitivity to the particular stress shows a significant overlap with the list of genes mutated in at least one of our 61 evolved lines. The table reports P-values adjusted for multiple testing using the false discovery rate control approach[3].

	Cell wall		Gyrase		Multiple	50s		30s		Folic acid	Aminoglycoside	
	**AMP**	**FOX**	**CPR**	**NAL**	**NIT**	**CHL**	**ERY**	**DOX**	**TET**	**TRM**	**TOB**	**KAN**
**No growth**	0.89	0.54	0.79	0.65	0.74	0.69	0.72	0.47	0.89	0.66	0.53	0.65
**Low growth**	0.02	0.30	0.08	0.17	0.14	0.09	0.16	0.20	0.01	0.26	0.26	0.21
**High growth**	0.09	0.16	0.13	0.19	0.13	0.22	0.13	0.33	0.10	0.08	0.21	0.15

Supplementary Table 5. Fraction of parallel evolving populations exhibiting different growth characteristics at the end of evolutionary experiment.

The table is based on the final cell densities. The laboratory evolution ended once at least 10 populations had showed growth or antibiotic concentration had reached its upper solubility limit. Growth categories were established as follows: i) ‘No growth’ was defined with an optical density (OD) under 0.05, ii) ‘low growth’ indicates lines in which OD didn’t reach at least 50% of the highest OD measured on the corresponding antibiotic, iii) ‘high growth’ consequently indicates lines where OD reached at least 50% of the highest OD.

Primer name	Sequence (5'-3')	Description
envZ-A	ccgaattcggttcgctggataatgc	*envZ* Arg411Gly mutation
envZ1-BF	tatccacgatacCctgcaca	*envZ* Arg411Gly mutation
envZ1-BR	attgtgcagGgtatcgtgga	*envZ* Arg411Gly mutation
envZ-C	ccggatccaacgccatcattgagca	*envZ* Arg411Gly mutation
envZ-D	cgcgcaggcttatctggtt	*envZ* Arg411Gly mutation
envZ-E	atgaccgcacgctgctgat	*envZ* Arg411Gly mutation
envZ2-BF	taatggtgcgcgTactgtcg	*envZ* Ala396Thr mutation
envZ2-BR	cgacagtAcgcgcaccatta	*envZ* Ala396Thr mutation
envZ3-A	ccggtaccgcgtagacacgaagattctc	*envZ* Val241Gly mutation
envZ3-BF	gtgacttCcccccgccatca	*envZ* Val241Gly mutation
envZ3-BR	atggcgggggGaagtcacga	*envZ* Val241Gly mutation
envZ3-C	ccggatccctatacgctggcgattatgc	*envZ* Val241Gly mutation
envZ3-E	gtgcgcgttgaggtcaaca	*envZ* Val241Gly mutation
marR-A	ccgaattctagccttgcatcgcattg	*marR* Val84Glu mutation
marR-BF	aaaggctgggAggaaaggtggg	*marR* Val84Glu mutation
marR-BR	aacctttccTcccagcctttac	*marR* Val84Glu mutation
marR-C	ccggatccagatagagtatcggctcg	*marR* Val84Glu mutation
marR-D	aacggcagcaacaccacca	*marR* Val84Glu mutation
marR-E	ttatgcggcggaacatcaa	*marR* Val84Glu mutation
mprA-A	ccgaattcaggagatccttctgactcg	*mprA* Leu136Arg mutation

Primer name	Sequence (5'-3')	Description
mprA1-BF	taactgccGgcatcaactctgg	*mprA* Leu136Arg mutation
mprA1-BR	ccagagttgatgcCggcagtta	*mprA* Leu136Arg mutation
mprA-C	ccggatccacgtatgcgtcatcggtt	*mprA* Leu136Arg mutation
mprA-D	ttagtgcgctggcctatgg	*mprA* Leu136Arg mutation
mprA-E	accagcacgtcgccttctt	*mprA* Leu136Arg mutation
mprA2-BF	ataacgatcTccgctgcctg	*mprA* Arg110Leu mutation
mprA2-BR	caggcagcggAgatcgttat	*mprA* Arg110Leu mutation
trkH-A	ccgaattctattggcggcttctcgac	*trkH* Thr350Lys mutation
trkH-BF	ccgggtcaaAgggcggtggc	*trkH* Thr350Lys mutation
trkH-BR	aggccaccgcccTttgaccc	*trkH* Thr350Lys mutation
trkH-C	aggaggcaatctcgcgcgtt	*trkH* Thr350Lys mutation
trkH-D	tatgtcttgctgaccgtcg	*trkH* Thr350Lys mutation
trkH-E	atgcggtgcacattggcga	*trkH* Thr350Lys mutation
phoQ-A	ccgaattccaccgagatgatcgtagctt	*phoQ* Gly384Cys mutation
phoQ-BF	gcacgttgcAcatcacctcg	*phoQ* Gly384Cys mutation
phoQ-BR	gaggtgatgTgcaacgtgct	*phoQ* Gly384Cys mutation
phoQ-C	ccggatcctgtgctctcagccaatctgc	*phoQ* Gly384Cys mutation
phoQ-D	ggtagttcacggaacggt	*phoQ* Gly384Cys mutation
phoQ-E	caacatcgcggatgccaa	*phoQ* Gly384Cys mutation
soxR-A	ccgaattcgcggaacattcgttgcaagt	*soxR* Leu139* mutation
soXR-BF	gcgaccgctGaggagaagaa	*soxR* Leu139* mutation

Primer name	Sequence (5'-3')	Description
soxR-BR	cttcttctCctcagcggtcg	*soxR* Leu139* mutation
soxR-C	ccggatccgttatcttcctgctccaggt	*soxR* Leu139* mutation
soxR-D	ctgctgcgagacataacc	*soxR* Leu139* mutation
soxR-E	aagatgcgtggcggacaa	*soxR* Leu139* mutation
Sce2	attaccctgttatcccta	pST76-A specific primer
T7	taatacgactcactataggg	pST76-A specific primer

Supplementary Table 6. Sequences of primers used for allele replacements.

Primers marked with A, C, BF, and BR were used to create homology regions by recombinant PCR for genomic integration of the suicide plasmids. Primers marked with D or E were homologous to flanking genomic regions, and were used for checking the allele replacements by PCR and sequencing. Capital letters in the BF and BR primers indicate the point mutation introduced in the gene.

**Antibiotic class excluded**	**Prediction performance against for antibiotic**											
	DOX	TET	KAN	TOB	CHL	ERY	CPR	NAL	FOX	AMP	TRM	NIT
ALL_drugs	0.84	0.71	0.74	0.80	0.76	0.55	0.77	0.88	0.74	0.74	0.75	0.80
30S	NA	NA	0.74	0.79	0.77	0.63	0.79	0.86	0.79	0.73	0.82	0.79
aminoglycoside	0.85	0.58	NA	NA	0.68	0.45	0.69	0.71	0.58	0.60	0.64	0.79
50S	0.83	0.72	0.89	0.87	NA	NA	0.79	0.87	0.75	0.77	0.80	0.79
Gyrase	0.84	0.73	0.76	0.87	0.85	0.58	NA	NA	0.78	0.78	0.71	0.81
Cell wall	0.82	0.67	0.73	0.80	0.77	0.57	0.81	0.95	NA	NA	0.74	0.98
Folic acid	0.85	0.76	0.76	0.80	0.76	0.54	0.77	0.89	0.76	0.76	NA	0.80

Supplementary Table 7. Predicting antibiotic resistance phenotypes from genome sequences.

The table shows changes in the prediction when a single antibiotic class was left out from the analyses. Prediction performance for each antibiotic based on the set of accumulated mutations was measured by the area under the receiver operating characteristic (ROC) curve (AUC). This gives an overall measure of accuracy by taking into account both true positive and false positive rates across all possible cutoffs of the prediction score. Random prediction gives an AUC of 0.5.

Supplementary References

1. Lazar V, *et al.* Bacterial evolution of antibiotic hypersensitivity. *Mol Syst Biol* **9**, 700 (2013).

2. Nichols RJ, *et al.* Phenotypic landscape of a bacterial cell. *Cell* **144**, 143-156 (2011).

3. Benjamini Y, Hochberg Y. Controlling the False Discovery Rate - a Practical and Powerful Approach to Multiple Testing. *J Roy Stat Soc B Met* **57**, 289-300 (1995).
